# Critical appraisal of the clinical utility of sublingual immunotherapy in allergy

**DOI:** 10.1016/j.conctc.2016.06.002

**Published:** 2016-06-18

**Authors:** S. Aissa, R. Ben Jazia, J. Ayachi, C. Ben Salem, A. Hayouni, A. Abdelghani, H. Ben Saad, M. Boussarsar

**Affiliations:** a Pulmonology Department, Farhat Hached University Hospital, Sousse, 4000, Tunisia; b Medical Intensive Care Unit, Farhat Hached University Hospital, Sousse, Tunisia; c Department of Clinical Pharmacology, Faculty of Medicine of Sousse, Tunisia; d Laboratory of Physiology, Farhat Hached University Hospital, Sousse, Tunisia

**Keywords:** Allergen specific immunotherapy, Sublingual immunotherapy, Allergy, Rhinoconjunctivitis, Asthma

## Abstract

Since it was introduced by Noon in 1911, allergen-specific immunotherapy or desensitization has been widely prescribed in the management of allergic diseases. Aimed at the etiology, it represents the only effective treatment for allergy.

The basic mechanisms of immunotherapy are becoming better understood and allow us to improve this technique in the future. The sublingual immunotherapy as an alternative to subcutaneous route has been widely studied. Several clinical trials confirmed that sublingual immunotherapy is efficient in reducing allergic respiratory symptoms. The sublingual immunotherapy reduces the risk of developing serious side effects due to desensitization.

We performed a literature review in order to remind the mechanisms of action and to demonstrate efficacy and tolerability of the sublingual immunotherapy in the treatment of allergic rhinoconjunctivitis and asthma and its impact on the quality of life.

## 1 Introduction to the management issues in the treatment of allergic patients

Asthma, like others allergic respiratory diseases, is a frequently encountered disease, and one that has a potentially serious impact on patient’s function and quality of life (QOL). In the Maghreb countries, the prevalence of asthma (3.4%–3.9%) is in the “low to moderate” range as stated by the latest AIRMAG survey [1]. Nonetheless, this prevalence is expected to rise as the populations become more urbanized and adopt a more ’Westernized’ lifestyle. On the other hand, asthma control is unacceptably poor in the Maghreb [1]. This could be changed by improved access to appropriate treatments, more proactive patient follow-up and better patient education. Furthermore, allergy is a highly prevalent clinical condition. It can be managed by the simple avoidance of the allergen, an anti-allergic such as antihistamines, a pathophysiological treatment such as corticosteroids or simply symptomatic treatment such as bronchodilators in case of asthma. Apart from these treatments, immunotherapy offers the advantage of altering the natural history of allergy by controlling the symptoms, reducing the consumption of these drugs and especially preventing asthma during the natural evolution of a rhinitis or better sustaining efficiency or preventing other sensitizations.

The allergen-specific immunotherapy is an effective therapeutic option in cases selected by rhinitis and/or by atopic asthma. Selection is mainly made by taking into account patients’ individual factors such as disease severity, efficacy of avoidance measures, pharmacological therapy and patients’ preferences. Allergen-specific immunotherapy is the process of administering increasing amounts of allergen(s) to allergic subjects in order to achieve hypo-sensitization that is to reduce the symptoms occurring during the natural exposure to the allergen itself. The history of allergen-specific immunotherapy began in the first years of the twentieth century. In fact, Noon aimed at achieving a vaccination against “airborne toxins,” and for this reason he chose, although unfunded, the subcutaneous route of administration [2]. Subcutaneous immunotherapy (SCIT) has been the conventional mode of therapy for patients with seasonal allergic rhino-conjunctivitis (ARC) and milder asthma that is unresponsive to pharmacotherapy [3]. With continued administration, it is expected that the treatment regimen will make the patient tolerant to the offending allergen and suppress future undesired responses to the allergen(s) through modulation of the patient’s immune system [4], [5]. However, this effective form of treatment is hindered by: ***1/***A prolonged injection schedule, ***2/***Patient non-compliance due to the frequent visits to the physician needed by the regimen and the delayed impact on the symptoms, ***3/***The discomfort associated with injections and, ***4/***The recognized risk of severe allergic reaction [3], [6], [7], [8].

During the same period, allergy management included new effective symptomatic and pathophysiologic drugs (β-agonists, corticosteroids, chromones, anti-leukotriene drugs) which demonstrated their efficiency on symptoms and QOL among allergic patients. In addition, there was a received idea stating that immunotherapy would not be effective particularly among older subjects.

Adding to these considerations, the SCIT, which is fundamentally criticizable, suffers from a poor acceptability, constraining young active patients to move to receive their injections. All these considerations have rapidly created a breach for the introduction of the sublingual immunotherapy (SLIT) in 1986 after a long – lasting oversight because of miscellaneous impediments.

SLIT involves desensitization by placement of the allergen extract under the tongue as an aqueous solution or as a dissolvable tablet for local absorption. SLIT was considered to be a better treatment modality associated with less severe systemic adverse effects and better compliance, but there is no demonstration that SLIT has additional mechanisms of action compared with SCIT. The use of SLIT provides an attractive option, for outpatient, home-based and self-administered therapy. The efficacy of SLIT has been demonstrated in children with allergic rhinitis and asthma, and the progression of atopy was reduced [9], [10]. SLIT seems to be more appropriate because of its safety, tolerable adverse effects, and good compliance [9], [10], [11]. However, recent data are conflicting. Indeed, the recent Cochrane meta-analysis for asthma [12] concluded that “lack of data for important outcomes such as exacerbations and QOL and use of different unvalidated symptom and medication scores have limited our ability to draw a clinically useful conclusion. Further research using validated scales and important outcomes for patients and decision makers is needed so that SLIT can be properly assessed as a clinical treatment for asthma”.

## 2 Review of the SLIT, pharmacology and mode of action

At optimal doses SLIT demonstrates effective. It could induce disease remission after discontinuation and may prevent new sensitization. These features are consistent with the induction of tolerance. In comparison with the subcutaneous immunotherapy, SLIT provides an additional local mechanism in oral mucosa and/or regional lymph nodes and leads to less systemic effects [8].

### 2.1 Pharmacology

The SLIT in suspension, as injectable allergen immunotherapy, is developed specially for every individual and prepared by the laboratory on a practitioner’s prescription and sent directly to the patient.

For tablets, two specialties are available: **i)** Grazax^®^ (75,000 SQ-T, oral Lyophilisat allergenic extracted standardized by pollen of grass of phléole of the meadows (Phleum pratense), having obtained a marketing authorization for the allergic rhinoconjunctivitis to pollens of grasses) is available in pharmacy and delivered on prescription [13] and **ii)** Oralair^®^ (allergenic extract of pollens: Dactylis glomerata L, Lolium perenne L, Anthoxanthum odoratum L, Poa pratensis and Phleum pratense L), which has a European marketing authorization [14].

These pharmaceutical forms allow an excellent reproducibility of the dose administered to every grip and do not impose the constraints of preservation in a cooled place as the solutions of allergens.

### 2.2 Mode of action

Information on the mechanisms of SLIT is less well-advanced. However, there is considerable knowledge regarding mechanisms of SCIT [15], [16]. For this reason, we aimed at exposing an overview of the SLIT mechanism of action.

### 2.3 Immunomodulation during the SLIT

It is now admitted that the efficiency of the desensitization is connected to the reorientation of the system Th2 *(Th for T helper cells)* towards the system Th1 and relying on the modulation of immune responses by specific regulating T-cells of the allergen [17].

The immune response in the atopic patient is characterized by a high production of type Th2 cytokines (IL4, IL5 and IL13, *IL for interleukin*) and by T-cells CD4^+^ *(CD for cluster of differentiation)* specific of the allergen [18], [19].

This cytokinic profile results in the secretion of specific immunoglobulin E (IgE) by B lymphocytes, as well as the recruitment and the activation of mastocytes, basophilic and eosinophilic cells which release mediators of the inflammation (histamine, tryptase, prostaglandins, leukotrienes, etc.) at the level of target organs [19], [20].

The SLIT, as well as SCIT, acts at the same time on humoral and cell mechanisms of the immune system involved in the allergic reaction [15], [21].

The SLIT leads to an increase in the production of allergen specific IgG4 and consequently to a limited impact on the production of the IgE. The correlation between the ratio IgE/IgG4 and the clinical efficiency of the immunotherapy was controversial [22], [23], [24], [25].

During the SLIT, we also observe a decrease in the recruitment of the pro-inflammatory cells (basophilic and eosinophilic) on the level of the skin, the nose, the eyes and the bronchial mucous membrane as well as an inhibition of their activation, leading to a significant reduction in the release of the pro-inflammatory mediators [26], [27].

The effect of the SLIT on the differentiation and the polarization of the allergen specific lymphocytic response TCD4+ is less well-documented than for the SCIT.

### 2.4 Role of the lymphocyte T regulator

Paradigm exchange of the specific immunotherapy aimed for a long time at redirecting the lymphocytic answers Th2 against the allergen towards an answer of type Th1 [28], [29].

The induction of allergen specific regulating T-cells (T Reg) inhibiting at the same time the answers Th1 and Th2 represents a hypothesis favored in the conception of new immunotherapeutic treatments nowadays [30], [31], [32].

Two categories of regulating cells were described according to their origin [33]. ***1/***T-cells natural Reg (CD4^+^, CD25^+^), characterized by the expression of the factor of transcription Foxp3, and deriving from the thymus [34]. ***2/***T-cells adaptive Reg which acquire their immunosuppressive activity after precursor’s activation T innocents (naive painters) of the peripheral blood [35]. Among the latters, a subgroup of lymphocytes Tr1 seems to play a major role. Lymphocyte Tr1 is dominant in the healthy subjects, tolerating the allergen. Their presence would be dominating in the initial phases of the allergen immunotherapy and the increase of these cells and their mediators (IL-10, Transforming growth factor β (TGF-β)) was noticed in the blood and in the peripheral organs as the nasal mucous membrane in patients desensitized.

During the allergen immunotherapy, modifications are also observed in lymphocytic B populations. The specific IgE of the allergen increases in the beginning of desensitization then tend to decrease secondarily. The synthesis of specific IgG4 and IgG1 of the allergen was able to be observed during the desensitization. The increase of the latter, as well as a serum factor blocking the fixation of the IgE in allergens, seemed to correlate in the clinical improvement of a group of asthmatic patients desensitized in the dust mites of house [36].

The IL-10, one of the key cytokines secreted by lymphocytes Tr1, plays a role in the orientation of the of B lymphocytes secretion profile towards the synthesis of IgG4 rather than that of the IgE.

As such, the IL-10 would be a powerful modulator of the tolerance, acting at the same time on the total IgE and on the specific IgE, with a final decrease of the ratio IgE/IgG4.

### 2.5 Specificity and pharmacodynamics of the buccal immune system

#### 2.5.1 General diffusion

The bottom of the tongue being much vascularized, blood vessels stream directly in the jugular vein. So, the small molecules administered by a sublingual way quickly reach the general circulation shunting the bowel or the liver [31], [32]. The obtained plasmatic concentration is maximal approximately 5 min after administration, with a global bioavailability of about 70% [37], [38].

#### 2.5.2 Local diffusion

The local environment in the mouth is regarded as a site of natural immune tolerance [15].

During the SLIT, the dendritic cell captures the allergen, in the site of the deposit on the buccal mucous membrane, in a few minutes. Then these cells run in the proximal lymph nodes, where they stimulate naive helper lymphocytes CD4^+^. The buccal mucous membrane contains a large number of dendritic cells of expressing receptors of high (RFceRI) and low (CD23) affinity for the IgE. These receptors may facilitate the capture of the allergen, and lead to the production of IL10, of TGF-β and an enzyme which catabolizes the tryptophan, leading to a decrease in the proliferation of lymphocytes T [39], [40].

The lymphocytic population and the local rates of IL10, TGF-β and interferon δ in this region of the mucous membrane appear to be more important than on the skin, suggesting a strong tolerogen power of this zone.

## 3 Efficacy studies and comparison to standard therapies

### 3.1 Efficacy on symptoms

During the last decade, many prospective trials have been made to study the efficacy of SLIT. These trials suffered from some limitations especially sample size and heterogeneity. This led to many meta-analyses and systematic reviews (Table 1).

**Table 1:** Summary results of meta-analyses of studies on sublingual immunotherapy efficacy.

Author [Reference] Journal Year	Study design	Studies (n)	Population	Patients (n)	Disease	Allergens	Main results
Wilson DR [41] Allergy 2005	Systematic review and meta-analysis	22	Adults and children	979	Allergic rhinitis	House dust mite, grass pollen, parietaria, olive, ragweed, cat, tree cupressus	Symptoms: −0,34 [−0.69, −0.15]^a^; p = 0.002 Medication requirements: −0,43 [−0.63, −0.23]^a^; p = 0.00003
Penagos M [42] Ann Allergy Asthma Immunol 2006	Meta-analysis	10	Children	484	Allergic rhinitis	Pollen, mite	Symptoms: 0,56 [1.01, 0.10]^a^; p = 0.02 Medication use: 0,76 [1.46, 0.06]^a^; p = 0.03
Calamita Z [43] Allergy 2006	Systematic review and meta-analysis	25	Adults and children	1706	Asthma	Pollen, mite, latex, dander, mould	64% of studies: significant reduction of asthma severity
Penagos M [44] Chest 2008	Meta-analysis	9	Children	441	Allergic asthma With/without rhinitis or rhino-conjunctivitis	Mite, holcus, P pretense dermatophagoides pteronyssinus, grass mix, europaea	Symptoms: −1.14 [−2.10, −0.18]^a^; p = 0.02 Medication use: −1.63 [−2.83, −0.44]^a^; p = 0.007)
Radulovic S [47] Allergy 2011	Cochrane systematic review	60	Adults and children	4589	Allergic rhinitis and/or conjunctivitis and/or asthma	ragweed, grass, parietaria, tree, house dust mite	Symptoms: −0.49 [−0.64, −0.34]^a^; p < 0.00001 Medication requirements: −0.32 [−0.43, −0.21]^a^; p < 0.00001
Calderon MA [42] Clinical & Exp Allergy 2011	Cochrane systematic review and meta-analysis	42	Adults and children	3958	Allergic rhino-conjunctivitis	–	Total ocular symptom scores: −0.41 [−0.53, −0.28]^a^; p < 0.00001
Lin SY [49] JAMA 2013	Systematic review	63	Adults and children	5131	Allergic rhino-conjunctivitis and Asthma	Dust mite, alternaria, grass mix, tree mix, birch, parietaria, ragweed, cat, olive, cedar, timothy grass	Moderate evidence supports that sublingual immunotherapy use decreases rhinitis or rhinoconjunctivitis symptoms, with 9 of 36 studies demonstrating greater than 40% improvement vs the comparator.
Wu YY [50] Zhonghua Nei Ke Za Zhi 2013	Meta-analysis	6	–	–	Allergic asthma		Asthma symptom scores: −0.89 [−1.36, −0.43]^a^; p = 0.0001 Asthma medication scores: −4.53 [−6.97, −2.08]^a^; p = 0.0001 FEV_1_: 0.19 [−0.02, 0.41]^a^; p = 0.078 Serum IgE level: 0.05 [−0.58, 0.69]^a^; p = 0.870
Tao L [51] Clin Respir J 2014	Meta-analysis	16	Adults and children	794	Allergic asthma	Mite	Symptoms: −0.74 [−1.26, −0.22]^a^; p = 0.006 Medication scores: −0.78 [−1.45, −0.11]^a^; p = 0.02
Normansell R [12] Cochrane Database Syst Rev 2015	Systematic review Meta-analysis	52	Adults and children	5077	Asthma with any allergen-sensitization pattern	House dust mite, pollen extract	Changes in inhaled corticosteroid use in micrograms per day: 35.10 [−50.21, 120.42]^b^; low-quality evidence Bronchial provocation: 0.69 [−0.04, 1.43]^a^; very low quality evidence

**FEV**_**1**_, First second forced expiratory volume; **IgE,** Immunoglobulin E.

a Data are standardized mean difference [95% confidence interval].

b Data are mean difference [95% confidence interval].

The first meta-analysis of SLIT, performed in 2005 by Wilson et al. [41], addressed only allergic rhinitis because the studies about allergic asthma were too scarce to perform a meta-analysis. It included 22 trials among 979 patients. Studies were very heterogeneous according to the allergen studies. Overall there was a significant reduction in both symptoms (standardized mean difference (SMD) [95% confidence interval (95%CI)], −0.34 [−0.69, −0.15]) and medication requirements (SMD [95%CI], −0.43 [−0.63, −0.23]) following immunotherapy.

Another meta-analysis of allergic rhinitis in pediatric patients, including 10 trials and 484 subjects, was performed by Penagos et al. [42]. It showed that SLIT was significantly more effective than placebo and demonstrated a significant reduction in both symptoms, (SMD [95%CI], 0.56 [1.01, 0.10]) and medication use (SMD [95%CI], 0.76 [1.46, 0.06]) after immunotherapy.

Regarding asthma, a meta-analysis by Calamita et al. [43] included 25 trials (either open or blinded) that involved 1706 adults and children. This meta-analysis found that SLIT was beneficial for asthma treatment albeit the magnitude of the effect was not very large. Moreover, it proved to be a safe alternative to the subcutaneous route.

Another meta-analysis by Penagos et al. [44] was also performed for asthma in pediatric patients. It included nine double-blind, placebo-controlled (DBPC) trials and 441 patients, and found a significant effect of SLIT on both asthma symptoms and rescue medication usage. However, it suffered from consistent heterogeneity due to various rating systems. The analysis demonstrated significant reduction in both symptoms after SLIT, (SMD [95%CI], −1.14 [−2.10, −0.18]) and medication use (SMD [95%CI], −1.63 [−2.83, −0.44]). Authors concluded that SLIT with standardized extracts induces a reduction in both symptoms and need for rescue medication among asthmatic children in comparison with placebo.

The meta-analyses mentioned above, pooled together all the allergens. Although SLIT appeared globally effective, the sub-analyses for single allergens provided uncertain results. Nevertheless, it is noteworthy that when a systematic evaluation of the efficacy was restricted to one specific allergen (house dust mite, HDM), the results remained positive [45]. Although authors detected a relevant inter-study heterogeneity, promising evidence of efficacy for SLIT, using mite extract in allergic patients suffering from allergic rhinitis and allergic asthma, was shown. It suggested that more data derived from high-quality studies based on large population are needed and should be corroborated by objective outcomes, mainly for allergic asthma.

In summary, these meta-analyses involved very heterogeneous trials, often without a proper sample size calculation. Publication biases and discrepancies in data collection were additional concerns [46]. Thus, they provided only suggestive evidence.

More recent systematic review by Radulovic et al. [47] included 60 studies among 4589 patients with rhino-conjunctivitis. Forty trials involved pollen SLIT. This publication confirms the efficacy of SLIT on standard evaluation criteria of allergic rhinitis, as well as on the symptom score and drug consumption. For eye symptoms, a meta-analysis by Calderon et al. [48] of 46 studies using sublingual (suspension or tablets) route showed that it was effective on the overall score of ocular symptoms and scores of individual symptoms (watery eyes, itchy eyes …).

A recent comprehensive meta-analysis by Lin et al. [49] included 63 studies with 5131 participants aged 4–74 years. Twenty studies (n = 1814 patients) involved only children. The risk of bias was medium in 43 studies (68%). Strong evidence supports that SLIT improves asthma symptoms, with 8 out of 13 studies reporting more than 40% of improvement *vs.* the comparator. Moderate evidence supports that SLIT use decreases rhinitis or rhino-conjunctivitis symptoms, with 9 of 36 studies demonstrating greater than 40% improvement *vs.* the comparator. Medication use, for asthma and allergies, decreased by more than 40% in 16 out of 41 studies of SLIT, with moderate grade evidence. Moderate evidence supports that SLIT improves conjunctivitis symptoms (13 studies), combined symptom and medication scores (20 studies), and disease-specific QOL (eight studies).

Another meta-analysis by Wu et al. [50] evaluated the efficacy of SLIT in patients with allergic asthma in order to provide reliable evidence for clinical application of SLIT. It showed that, compared with control group, SLIT could significantly reduce asthma symptom scores (SMD [95%CI], −0.89 [−1.36, −0.43]) and asthma medication scores (SMD [95%CI], −4.53 [−6.97, −2.08]), but neither first second forced expiratory volume (FEV_1_) (SMD [95%CI], 0.19 [−0.02, 0.41]; p = 0.078), nor serum IgE levels (SMD [95%CI], 0.05 [−0.58, 0.69]; p = 0.870).

A meta-analysis by Tao et al. [51] was performed to investigate the clinical efficacy and safety of SLIT for allergic asthma. Sixteen randomized DBPC trials, with a total of 794 patients, suggested that SLIT significantly reduces both symptom scores (SMD [95%CI], −0.74 [−1.26, −0.22]) and medication scores (SMD [95%CI], −0.78 [−1.45, −0.11]) compared with placebo. SLIT clinical response was more efficient in mite sensitive asthmatics. Prolonged duration of treatment for more than 12 months proved to bring no additive effects. Immunotherapy has also improved the skin prick test, but there was no consistent effect on FEV_1_, antigen specific sIgG4, and sIgE. The relative risk of adverse effects was 2.23 (p = 0.01).

In the 2015 systematic review and meta-analysis by Normansell et al. [12] including 5077 asthmatics, the SLIT effects on changes in inhaled corticosteroid use and bronchial provocation were qualified, respectively, as low and very low quality evidence.

### 3.2 Impact of SLIT on the natural history of respiratory allergy

Allergy is not a static entity, but it could evolve in its clinical presentation over time. In some patients, this entity was addressed to as the so called “atopic march”. Thus it is well known that allergic rhinitis often precedes asthma [52], [53], [54], [55], [56], [57] or the development of bronchial hyperreactivity [58], [59]. Another aspect of the natural history of respiratory allergy is the trend to develop new sensitizations over time [59].

The effect on natural history of respiratory allergy was described with SCIT more than 40 years ago [60], [61], [62], [63], [64], [65], [66], [67]. Because the first clinical trials with SLIT aimed at demonstrating efficacy and safety, the disease modifying effects have only been addressed over the past 10 years.

The first study showing that SLIT may prevent the onset of asthma was published in 2004 [68]. After three years, development of asthma was 3.8 (95%CI, [1.5, 10]) times more frequent in the control subjects. These results were confirmed in another study that demonstrated that asthma occurrence was reduced from 30% in controls to 1.5% in SLIT group, with a number of four cases to be treated. Other studies demonstrated that SLIT was also capable of preventing the onset of bronchial hyperreactivity to methacholine [69], [70].

Other randomized controlled trials suggested a preventive effect on new skin sensitizations with SLIT. In 511 patients randomly submitted to SLIT or drugs alone, Marogna et al. [71] demonstrated that new sensitizations appeared in 38% vs. 5.9% respectively in control vs. SLIT patients.

Another clinical effect of SLIT on the natural history of allergy is the long-lasting effect. Indeed, several studies largely demonstrated that a 3–4 years course of SLIT represents the best combination of clinical efficacy and a long term effect was observed up to seven years after discontinuation [11], [72], [73], [74].

### 3.3 SLIT vs. SCIT

As stated before, many systematic reviews and meta-analyses on allergen immunotherapy have been conducted in recent years (Table 1). However, direct comparisons of subcutaneous and sublingual immunotherapy were rare. This may be the result of the reluctance of manufacturers to conduct such studies, or rather because of the methodological aspects of double blinding.

In 2009, the World Allergy Organization (WAO) reported a review of randomized controlled trials dealing with a comparison between SCIT and SLIT [8]. In fact, only two studies were double-blind double-dummy [75], [76]. One of these, carried out by Khinchi et al. [76], was placebo controlled and demonstrated that symptoms and medication use were reduced by about one third in the SLIT group and by a half in the SCIT group. Overall analyses concluded to no difference between the two treatments. The four remaining trials [77], [78], [79], [80] were all conducted in an open fashion.

In another systematic review conducted by Chelladurai et al. [81], eight studies have been included. All of them were head to head but only three included controls [76], [78], [82]. This review also included the well done study by Khinchi et al. [76]. Authors concluded that the review provides low-grade evidence showing that SCIT is superior to SLIT in asthma symptom reduction and moderate-grade evidence showing reduction in rhino-conjunctivitis [81].

A more recent and innovative systematic review by Detzke et al. [83] included respectively 17 placebo controlled randomized controlled trials for SCIT and 11 for SLIT. Only one study was head to head trial (the one by Khinchi et al. [76]). The originality of this review was to proceed to indirect comparisons of placebo controlled studies respectively with SLIT or SCIT. The advantage is to avoid the huge proportion of drop-outs in head to head studies as in the one by Khinchi et al. [76] or all the other studies included in the review by Chelladurai et al. [81]. The indirect comparison favored SCIT over SLIT in terms of symptom score but not in terms of the combined symptom medication or QOL. Authors concluded that despite evidence of efficiency of both SLIT and SCIT, the superiority of one over the other could not be proven through indirect comparison [81].

From another inception, a study carried out in Asia in 2011 showed that SLIT has been establishing its role for allergic rhinitis [84]. Long-term use of SLIT could alter immunologic profiles. SLIT, as well as SCIT, also prevents poly-sensitization and development of asthma. It also demonstrated that there seems to be less risk of severe or fatal adverse events than in SCIT [85].

Since the consensus of the WAO in 1988 [8], several meta-analyses were conducted to prove the efficacy of SLIT, and are regularly updated by different organizations and scientific societies (ARIA: Allergic Rhinitis and its Impact on Asthma, EAACI: European Academy of Allergy and Clinical Immunology, AAAAI: American Academy of Allergy, Asthma & Immunology).

## 4 Safety and tolerability

While large scale randomized DBPC trials, position papers, and meta-analyses have emphasized the efficacy of SLIT, safety has been less addressed [8], [46], [86], [87]. One of the suggested advantages of SLIT over SCIT is its greater safety, which would allow its administration outside the medical field. It has been demonstrated that while both are equally efficient, SLIT leads to “less” and “less severe” adverse events than SCIT [75], [76], [77], [78], [79], [80], [88]. Compared to SCIT, SLIT is generally considered to have a better safety profile. In SLIT, most reactions are local and transient and do not lead to interruption or cessation of treatment [89].

In a comprehensive review of 104 articles dealing with SLIT, information on safety and tolerance were provided by 66 studies conducted on 4378 patients receiving about 1,181,000 SLIT doses [89]. The amount of information on the adverse events in these studies varied greatly, ranging from general summary statements, such as “no relevant side effects,” to a detailed analysis of the adverse events [90]. Because of the vast heterogeneity in classifying and reporting SCIT adverse events, some efforts have been made to develop a uniform severe reactions grading system [90].

SLIT adverse events were frequently local (75%) and 0.06% of doses administered were classified as severe reactions. The majority of these reactions were gastrointestinal symptoms, rhino-conjunctivitis, urticaria, or some combination of these symptoms [91].

The SLIT comprehensive review [89], reported neither fatalities nor anaphylactic reactions. However, there were 14 probable SLIT-related serious adverse events (SAE) in 3984 patients treated with a total of 1,019,826 doses in 58 studies. This represents 1.4 SAEs per 100,000 SLIT doses and one SLIT-related SAE per 384 treatment years or 285 patients. Asthmatic reactions were the most common SLIT-related SAE. Abdominal pain and vomiting (n = 3), uvula edema (n = 1) and urticaria lasting 48 h were also reported [89].

Subsequent to these data, some cases of severe adverse events associated with anaphylaxis were reported with SLIT [92], [93], [94], [95].

## 5 Patient focused perspectives such as QOL, patient satisfaction and acceptability

### 5.1 Adherence

Adherence (acceptance and persistence) to treatment is often a problem in chronic conditions, particularly with SLIT in asthma and rhinitis where treatment has no immediate impact on symptoms. Subcutaneous and sublingual approaches differ significantly in terms of determinants of adherence (related medical/hospital administration for SCIT, by the patient for the SLIT), although few data are published.

A multicenter observational study by Lombardi et al. [96] was specifically designed to provide a quantitative measure of SLIT adherence. Eighty-six patients with allergic rhinitis, asthma, or both were treated by SLIT. Adherence was about 97% for all patients. Omitted doses were reported in 11 patients, with most of who postponed one or two doses because of concurrent illness or forgetfulness. One patient skipped multiple doses because of work schedule [96].

In a randomized, 4-year open study of 511 patients with allergic rhinitis, asthma, or both caused by various allergens, adherence to SLIT over the 3-year period was excellent (>80%), good (from 60% to 80%) and poor or insufficient (<60%), respectively in, 72%, 18% and 10% of the 271 included patients [97].

In another study, Passalacqua et al. [98] demonstrated that at three months, 85% of subjects had a compliance rate >75% (69% of them adhered >90%). At six months, 84% had a compliance rate >75% (66% of them adhered >90%) [98].

Canonica et al. [99] demonstrated that the cost of allergen given via sublingual route was found to be high. Nevertheless, this was balanced by a reduced need for medical and nursing time and by the absence of the cost of injections. Taking into account all these aspects, the global cost of SLIT is even lower than that of SCIT [99].

A recent study by Incorvaia et al. [100] concluded that after the first optimistic reports in recent years on a very high adherence to SLIT, it became apparent that SLIT is plagued by the same issue of low adherence that affects drug treatment. The degree of adherence is particularly low in the third year of SLIT, and this prevents the disease-modifying effect on the natural history of allergy from being achieved, the occurrence of which ensures the long-lasting clinical benefits and the consequent pharmaco-economic advantages [100]. The search for optimal adherence is a question of balancing a number of factors. Improving adherence to SLIT is a major goal, and the recent studies suggest that patient education, accurate monitoring during treatment and possibly technology-based tools are interventions that are likely to meet such a need [100].

### 5.2 Quality of life

In a randomized double blind study, authors demonstrated that SLIT added to a combination of grass and olive pollen extracts was better tolerated when given directly in the maintenance phase [101]. These results agree with those reported by a previous multicenter study conducted without up dosing [102]. It demonstrates a clinically significant improvement in QOL with rhinitis overtime of 1.42 (>0.5 the smallest clinically relevant value, as stressed by Guyatt and Jaescheke [103] and proposed in the questionnaire of Juniper et al. [104], [105].

In another study by Ciprandi et al. [106], the effect of SLIT on QOL was clinically relevant to activities, practical problems, and nasal and ocular symptoms. This is interesting as these are the aspects that have proved to be affected most in allergic rhinitis [106].

In a study by Morris et al. [107], SLIT was demonstrated to be effective in reducing symptoms and improving QOL after a four-months of treatment. This improvement was most prominent in activity, non-nose/eye symptoms, nasal symptoms, and emotional domains. Improvement in the sleep domain of the rhino-conjunctivitis quality of life questionnaire (RQLQ) was also observed, but did not reach statistical significance. This improvement was sustained and demonstrated again at 10–12 months of treatment. Taking into account the high compliance rates and the La Crosse Method, it is expected that the improvement achieved is likely to be sustained and possibly expanded beyond the first year of ongoing treatment [107].

In another study by Wise et al.[108], paired mini-RQLQ results revealed statistically a significant improvement on 12 of 14 domains assessed by this questionnaire [108]. Improvements in the impact on regular and recreational activities, sleep, nose rubbing and nose blowing, stuffy nose and runny nose, itchy eyes, sore eyes, watery eyes, thirst, and tiredness were seen. Total mini-RQLQ score also showed statistically significant improvement, with a decrease in mean score from 27.8 before initiation of SLIT to 12.6 during the maintenance phase of therapy.

## 6 Conclusion

Sublingual allergen specific immunotherapy, an aetiology-based treatment for respiratory allergic diseases, is gaining an accumulating evidence of efficacy and safety compared to the subcutaneous classic route of administration. In some European countries, it is currently used more frequently. When administrated at high doses regularly for at least three consecutive years, SLIT could demonstrate some advantages over SCIT: ***1/***Better tolerance with only local reactions, ***2/***Better compliance and better QOL. The heterogeneity of trials involved in meta-analyses, the use of different allergens, the publication biases and discrepancies in data collections provided only suggestive evidence of the superiority of SLIT over SCIT. Further well-designed randomized controlled trials are needed to support this evidence base for clinical decision-making. These trials would focus especially on optimal patient selection, dosage, protocol and treatment duration.

## Conflict of interest

None.
